# Digital Devices for Arrhythmia Detection: What Is Still Missing?

**DOI:** 10.1002/clc.70074

**Published:** 2024-12-23

**Authors:** Naoya Kataoka, Teruhiko Imamura

**Affiliations:** ^1^ Second Department of Internal Medicine University of Toyama Toyama Toyama Japan

**Keywords:** atrial fibrillation, monitoring, wearable device

To the Editor,

Various digital devices have been developed to detect atrial fibrillation (AF). Manninger and colleagues demonstrated that intermittent electrocardiogram (ECG)‐based digital devices are increasingly being integrated into clinical practice, whereas photoplethysmography (PPG)‐based devices are less commonly utilized for the diagnosis and screening of AF [1].

In the three proposed clinical scenarios—(1) symptomatic low‐risk patients, (2) asymptomatic high‐risk patients, and (3) symptomatic high‐risk patients—many clinicians demonstrated a preference for diagnostic tools other than PPG‐based devices [1]. The authors highlighted this trend and emphasized the need for enhanced education regarding novel PPG‐based digital technologies. However, ECG‐based tools, which provide greater diagnostic accuracy than PPG‐based devices, may be more suitable in scenarios (1) and (3), where patients already exhibit clear symptoms of AF.

The debate over the type of device (PPG‐based vs. ECG‐based) may not be the most critical issue at present. While PPG‐based devices demonstrate high accuracy in AF screening [2], the duration of arrhythmia monitoring is arguably more significant. Devices that impose minimal burden on patients are likely to be preferred for prolonged monitoring.

The clinical implications of aggressive intervention for AF detected by wearable devices remain uncertain. The prevalence of AF in the general population is quite low (approximately 1%) [2], and among those diagnosed, only 30% experience adverse outcomes such as thromboembolism or heart failure. Consequently, the number needed to treat to achieve a meaningful clinical benefit from wearable devices is exceptionally high [3]. Therefore, optimizing patient selection for the implementation of PPG‐based devices warrants greater consideration.

## Ethics Statement

The authors have nothing to report.

## Conflicts of Interest

The authors declare no conflicts of interest.

## Data Availability

The authors have nothing to report.

## References

[1] M. Manninger , D. Zweiker , T. Hovakimyan , et al., “Physician Preferences in Using Novel Digital Devices for the Management of Atrial Fibrillation‐A DAS‐CAM III Survey,” Clinical Cardiology 47 (2024): e24331. 39582318 10.1002/clc.24331 PMC11586575

[2] T. Pereira , N. Tran , K. Gadhoumi , et al., “Photoplethysmography Based Atrial Fibrillation Detection: A Review,” NPJ Digital Medicine 3 (2020): 3. 31934647 10.1038/s41746-019-0207-9 PMC6954115

[3] S. A. Lubitz , C. Moser , L. Sullivan , et al., “Atrial Fibrillation Patterns and Risks of Subsequent Stroke, Heart Failure, or Death in the Community,” Journal of the American Heart Association 2, no. 5 (2013): e000126. 24002369 10.1161/JAHA.113.000126 PMC3835216

